# Depressive Symptoms Moderate the Association Between Lifetime Discrimination and Inhibition Among Older Adults

**DOI:** 10.1093/geroni/igab046.2643

**Published:** 2021-12-17

**Authors:** Regina Wright, Desiree Bygrave

**Affiliations:** 1 University of Delaware, University of Deleware, Delaware, United States; 2 Benedict College, Benedict College, South Carolina, United States

## Abstract

Discrimination has been identified as a potentially modifiable environmental stressor that reduces cognitive function. As the burden of discrimination can extend from early to late life, understanding its role in cognition in late life is critical. Further, understanding the potential moderating influence of depressive symptoms, which are common among older adults, on the linkage between discrimination and cognition, may provide further insight into the potential patterns of psychosocial stress and negative affect that may promote cognitive decline and dementia. Thus, we sought to examine whether depressive symptoms moderate linear relations of lifetime discrimination to cognitive function in the domains of visuospatial, verbal, and working memory, executive function, and psychomotor ability, adjusting for age, sex, race, and education. Participants were 165 older adults (34% male) with a mean age of 68.43y. Participants completed a health screening, a battery of cognitive tests, a psychosocial assessment, and cardiovascular testing relevant to the larger study. Linear regression results showed a significant interaction between lifetime discrimination and depressive symptoms (p<.05) related to the Stroop interference score, a measure of inhibition. A probe of the interaction showed that greater lifetime discrimination was associated with better inhibition among participants with fewer depressive symptoms. This paradoxical finding is consistent with scant research that shows exposure to discrimination may heighten performance, and is more common among individuals that have achieved more, both educationally and vocationally. Greater depressive symptomatology may reduce this paradoxical association. Future research should explore this question both longitudinally and in a larger sample.

